# Pyrolysis Kinetic Study and Reaction Mechanism of Epoxy Glass Fiber Reinforced Plastic by Thermogravimetric Analyzer (TG) and TG–FTIR (Fourier-Transform Infrared) Techniques

**DOI:** 10.3390/polym12112739

**Published:** 2020-11-18

**Authors:** Yuanhua Qiao, Oisik Das, Shu-Na Zhao, Tong-Sheng Sun, Qiang Xu, Lin Jiang

**Affiliations:** 1 School of Mechanical Engineering, Nanjing University of Science and Technology, Nanjing 210094, China; 18801592308@163.com (Y.Q.); zhaoshunafairy@outlook.com (S.-N.Z.); 314101002278@njust.edu.cn (T.-S.S.); xuqiang@njust.edu.cn (Q.X.); 2 Structural and Fire Engineering Division, Department of Civil, Environmental and Natural Resources Engineering, Luleå University of Technology, 97187 Luleå, Sweden; oisik.das@ltu.se

**Keywords:** glass fiber reinforced plastic, pyrolysis, model-free, model fitting, fourier-transform infrared

## Abstract

TG–FTIR combined technology was used to study the degradation process and gas phase products of epoxy glass fiber reinforced plastic (glass fiber reinforced plastic) under the atmospheres of high purity nitrogen. The pyrolysis characteristics of epoxy glass fiber reinforced plastic were measured under different heating rates (5, 10, 15, 20 °C min^−1^) from 25 to 1000 °C. The thermogravimetric analyzer (TG) and differential thermogravimetric analyzer (DTG) curves show that the initial temperature, terminal temperature, and temperature of maximum weight loss rate in the pyrolysis reaction phase all move towards high temperature, as the heating rate increases. Epoxy glass fiber reinforced plastic has two stages of thermal weightlessness. The temperature range of the first stage of weight loss is 290–460 °C. The second stage is 460–1000 °C. The above two weight loss stages are caused by pyrolysis of the epoxy resin matrix, and the glass fiber will not decompose. The dynamic parameters of glass fiber reinforced plastic were obtained through the Kissinger-Akahira-Sunose (KAS), Flynn–Wall-Ozawa (FWO) and advanced Vyazovkin methods in model-free and the Coats–Redfern (CR) method in model fitting. FTIR spectrum result shows that the main components of the product gas are CO_2_, H_2_O, carbonyl components, and aromatic components during its pyrolysis.

## 1. Introduction

Glass fiber reinforced plastic generally refers to a composite material with a glass fiber reinforced polymer resin matrix, which is widely used in aviation, aerospace, weapons, chemical, construction, and marine fields considering its good performance in mechanical properties, thermal insulation properties, and corrosion resistance [1,2,3,4,5,6,7,8]. In the military field, epoxy glass fiber reinforced plastic is widely employed to replace traditional steel and alloy components. When faced with extremely high heat flux (megawatt level), the temperature inside the material and at its surface can increase sharply, and its ablation behavior can influence its thermal stability and characteristics. At present, most research studies on thermal analysis of glass fiber reinforced plastics focus on reinforcement preparation and mechanical modification, and there are only few studies on the thermal analysis of glass fiber reinforced plastics. Additionally, most of the existing thermal analysis studies only focus on the use of the model-free method to solve the dynamic parameters of glass fiber reinforced plastics, without involving methods to solve pyrolysis mechanism kinetically.

Yun et al. [9] used TGA coupled with a pyrolyzer to monitor the thermal behavior and evolved gas of epoxy glass fiber reinforced plastic from 500 to 900 °C under non-isothermal conditions. Results show that the main weight loss interval of epoxy glass fiber reinforced plastic ranges from 230 to 430 °C. Then, the Friedman method was used to calculate the kinetic parameters of the thermal decomposition of epoxy glass fiber reinforced plastic, and results show that its activation energy ranges from 41.4 to 78.4 kJ/mol. The main components of the evolved gas are carbon monoxide from the ether and carbonyl decomposition, and hydrogen from the cleavage of the aromatic ring. Zhang et al. [10] used a simultaneous thermogravimetric analyzer-differential scanning calorimetry (TG-DSC) analyzer to study the pyrolysis characteristics of epoxy glass fiber reinforced plastic under different heating rates and different carrier gas atmospheres. Then, the Kissinger and the Flynn–Wall–Ozawa methods were employed to obtain the apparent activation energy of each conversion during the pyrolysis process. Results show that the thermal decomposition process of epoxy glass fiber reinforced plastic under air atmosphere can be divided into two stages, while the thermal decomposition of glass fiber reinforced plastic under nitrogen atmosphere has only one stage, and its thermal stability shows a gradually increasing trend during pyrolysis. Zheng et al. [11,12] used the SDT Q600 synchronous thermal analyzer to study the pyrolysis characteristics of epoxy glass fiber reinforced plastic samples under unirradiated and different irradiation doses, and by using the Friedman and FWO methods, the activation energy was calculated under different material irradiation conditions. Results show that the maximum thermal decomposition weight loss rate and activation energy of epoxy glass fiber reinforced plastic increased with the increase in irradiation dose. In all the above studies, model-free methods were used to solve the kinetic parameter activation energy of epoxy glass fiber reinforced plastics, and there was a lack of a model fitting method to solve its pyrolysis reaction model. In addition, most of the above studies did not involve the study of the chemical reaction mechanism of epoxy glass fiber reinforced plastics. Other researchers focused on the pyrolysis behavior of pure epoxy or glass fiber reinforced plastics separately. Shi and Bao [13] investigated the effect of pyrolysis time and temperature on recycled glass fiber reinforced plastic and explored its optimum decomposition conditions with superheated steam. Results show that methods using superheated steam provided resin removal efficiencies above 80 wt % with the glass fiber kept. Chen and Yeh [14] studied the pyrolysis kinetics of pure epoxy resins under four different heating rates in nitrogen with TG. Results show that the averaged activation energy is around 200 kJ mol^−1^, and the activation energy can increase with pyrolysis conversion monotonously. Nakagawa et al. [15] studied the pyrolytic behaviors of thermosetting epoxy samples with the high-resolution pyrolysis gas chromatography method. Samples, epoxy prepolymers and epoxy resin samples cured with and without curing agents, were used. Then, the evolved gas of epoxy and the effects of its curing agents were identified.

In this study, solving the kinetic mechanism of glass fiber reinforced plastics through model fitting methods and exploring its chemical reaction mechanism through evolved gas analysis are what this research will add to the research area of material pyrolysis. TG and TG–FTIR methods were used to obtain the thermogravimetric curves of the epoxy glass fiber reinforced plastics at different heating rates and the infrared spectra of evolved gas during the pyrolysis process with 20 °C min^−1^ heating rates. Then, model-free and model fitting methods were employed to determine the pyrolysis kinetic reaction model and kinetics of epoxy glass fiber reinforced plastics. By analyzing the infrared spectrum of evolved gas, the chemical reaction mechanism of epoxy glass fiber reinforced plastics can be obtained. This article can provide more guidance information for the study of the thermal stability and combustion behavior of epoxy glass fiber reinforced plastics.

## 2. Experimental

The epoxy glass fiber reinforced plastic samples used in this study were produced by Weihai Guangwei Composite Materials Co., Ltd. (Weihai, Shan Dong, China). The epoxy glass fiber board was made of G20000 glass fiber epoxy prepreg composed of E-type glass fiber and 6509 epoxy resin by a molding method, in which the glass fiber content is 67%. The specific curing process was described as follows: at room temperature, the prepreg was heated to 80 °C with a rate of 2 °C/min. After 5 to 10 min of heat preservation, it was pressurized, and the pressure was set to 2 Mpa. After the prepreg was kept under heat with 15–25 °C/min, it was heated to 125 °C at a rate of 2 °C/min. After holding for 120 min, it was naturally cooled to room temperature. In the TGA experiment of this study, a sample of epoxy glass fiber board was ground into powder of 2 mg for each testing. A NETZSCH-STA-449C synchronous thermal analyzer manufactured by Netzsch Co., Ltd. (Exton, PA, USA) was used in a nitrogen atmosphere (nitrogen with 20 mL min^−1^ flow rate). The TGA crucible holding sample is Al_2_O_3_ type. The sample was heated in the TGA from 25 to 1000 °C with 5, 10, 15, and 20 °C min^−1^ heating rates. In the TGA-FTIR experiment of this study, firstly, the TG–FTIR measurement was conducted on a STA8000 simultaneous thermal analyzer (Perkin-Elmer, Waltham, MA, USA) coupled with a FTIR spectrophotometer (Perkin-Elmer, Waltham, MA, USA). In the following measurements, a sample of epoxy glass fiber that weighed 2 mg was heated from 20 to 800 °C under nitrogen atmosphere with a heating rate of 20 °C/min. The formed volatile compounds were immediately transferred to the gas cell of the coupled FTIR spectrophotometer through a connecting pipe. The spectral region was 500–4000 cm^−1^ while the FTIR resolution was set at 8 cm^−1^.

## 3. Kinetic Method

The mass loss with time or temperature can be recorded by TGA, under the condition that the sample was heated linearly within a certain temperature range. In general, we can use the function of conversion rate to express the thermal weight loss. The conversion rate at a certain moment/temperature can be defined as the ratio of the weight of the sample that has reacted to the unreacted weight at that moment, that is,

(1) $$\alpha =(M_{0}-M)/(M_{0}-M_{\infty })$$

In Equation (1), *α* is the conversion rate of the sample at a certain time, and M, $M_{0}$, and $M_{\infty }$ are the mass of the sample at a certain time during the reaction process, at the beginning of the reaction, and at the end time of the reaction. We can use the change of conversion rate with time to express the chemical reaction rate in the thermal decomposition kinetics. The chemical reaction rate is composed of two parts. One is the reaction mechanism function regarding the conversion rate, and the other part is the Arrhenius equation, which is the empirical formula for the relationship between the chemical reaction rate constant and temperature change. Therefore, the chemical reaction rate can be expressed as

(2) dα/dt=β(dα/dT)=k(T)f(α)

In the above formula, α is the conversion rate, *β* is the heating rate, *T* is the sample temperature, *t* is time, *k*(*T*) is the chemical reaction rate constant, and *f*(*α*) is the function of the reaction mechanism. Combining Equation (2) with the Arrhenius formula *k* = *A*exp(−*Ea*/*RT*) can be obtained:

(3) dα/dt=Aexp(−Ea/RT)f(α)

where *A* is the pre-exponential factor, *Ea* is the activation energy of the chemical reaction and *R* is the gas constant 8.314 J·(mol·K)^−1^. Among the parameters described above, we call the activation energy, the pre-exponential factor, and the reaction mechanism function kinetic triplets. By calculating and analyzing the kinetic triplets, relevant conclusions about the thermal stability and thermal decomposition mechanism of the sample can be obtained accordingly. Methods for solving the three kinetics factors can be roughly divided into the model-free method and the mode fitting method.

### 3.1. Model-Free Method

The model-free method does not need to consider information about the specific chemical reaction model. It is assumed that the reaction rate for a certain conversion rate is only related to the temperature, which is also called the isoconversional method. Due to the different assumptions of the calculation method itself, this will cause calculation errors. In this paper, three commonly used methods, the KAS method, the FWO method, and the advanced Vyazovkin method are used for kinetic calculations of epoxy glass fiber reinforced plastic pyrolysis. The KAS method is an improved method compared to the existing Coats–Redfern approximation method [16,17]. The expression of the KAS method can be written as:

(4) $$\ln(\frac{\beta }{T^{2}})=\ln\frac{AR}{E_{a}g(\alpha )}-\frac{Ea}{RT}$$

where *g*(*α*) is the integral form of the inverse of *f*(*α*), which can be expressed as:

(5) $$g(\alpha )\approx \frac{A}{\beta }\int_{0}^{T}e^{-\frac{Ea}{RT}}dt$$

According to the expression of the KAS method, the activation energy *Ea* and the pre-exponential factor *A* can be calculated by fitting ln(*β*/*T*^2^) and 1/*T* with different heating rates. In the case of *Ea*/*RT* > 13, the activation energy calculated by the KAS method can achieve a good calculation. In addition, based on Doyle’s hypothesis, Flynn, Wall [18] and Ozawa [19] jointly proposed the FWO method. As an integral method, the FWO method does not need to introduce differential data on thermal weight loss [20,21,22,23,24,25], and its expression can be written as follows:

(6) $$\mathrm{lg}\beta =\mathrm{lg}\frac{AEa}{Rg(\alpha )}-2.315-0.4567\frac{Ea}{RT}$$

From Equation (6), it can be concluded that under the conversion *α*, *g*(*α*) is a constant value. At this assumption, lg*β* and 1/*T* have a linear relationship. From the slope (−0.4567*Ea*/*RT*), the activation energy *Ea* at each conversion can be obtained. Vyazovkin et al. [26,27,28] found a new isoconversional method, which is based on the integral method and can help obtain higher accuracy. In the advanced Vyazovkin kinetic calculations, there are the following integral equations:

(7) $$I(E_{\alpha },T_{\alpha })\approx \int_{0}^{T_{\alpha }}\exp(-\frac{Ea}{RT})dT$$

(8) $$I=\frac{Ea}{R}p(x)$$

In Equation (8), *p*(*x*) is the temperature integral formula, which is difficult to solve numerically. Hence, the value of *p*(*x*) has to be obtained by numerical approximation. Many researchers have already raised many approximate solutions during kinetic calculation. In this study, the following formula is used to find the approximate value of *p*(*x*) [29]:

(9) $$p(x)\approx \frac{\exp(-x)}{x}\times (\frac{x^{5}+40x^{4}+552x^{3}+3168x^{2}+7092x+4320}{x^{6}+42x^{5}+630x^{4}+4200x^{3}+12600x^{2}+15120x+5040})$$

Then, through calculating the minimum value of Equation (10), the value of the activation energy at each conversion can be obtained, that is,

(10) $$\Omega (Ea)=\sum_{i=1}^{n}\sum_{j\neq 1}^{n}\frac{I(Ea_{\alpha },T_{\alpha .i})\beta_{j}}{I(Ea_{\alpha },T_{\alpha ,j})\beta_{i}}$$

Through repeated iterative calculations from Equations (7) to (10), a set of relatively precise activation energies can be obtained at every chosen conversion rate.

### 3.2. Model Fitting Methods

Generally, when the pyrolysis process can be described by the kinetic model, the model can be figured out using the model fitting method. Obviously, the accuracy of the obtained kinetic parameters depends on the matching degree of the selected response model. Table 1 [30] shows the commonly used pyrolysis models in kinetics model fitting area. As a result of the non-isothermal TG tests, the model fitting method was used to fit the α–T curves obtained from the test to classical reaction models as listed in Table 1. After that, a series of activation energies and pre-exponential factors with respect to each classic model can be obtained [31]. Among the model fitting methods, the Coats–Redfern (CR) method [32,33] can be viewed as one of the most common and popular methods, and the expression can be described as follows:

(11) $$\ln\frac{g(\alpha )}{T^{2}}=\ln(\frac{AR}{\beta Ea}[1-(\frac{2RT}{Ea})])-\frac{Ea}{RT}$$

In general, when $\frac{Ea}{RT}\gg 1$, $1-\frac{2RT}{Ea}\approx 1$, Equation (11) can be reduced to the form of Equation (12):

(12) $$\ln\frac{g(\alpha )}{T^{2}}=\ln(\frac{AR}{\beta Ea})-\frac{Ea}{RT}$$

According to Equation (12), for each *g*(*α*) model, ln[*g*(*α*)/*T*^2^] and 1/*T* have a linear relationship. The slope and intercept of the straight line can be used to directly solve the chemical reaction to obtain the activation energies and pre-exponential factors. We can regard the reaction model with the best numerical fitting linearity among all classical models listed in Table 1 as the kinetic reaction model of epoxy glass fiber reinforced plastic pyrolysis.

## 4. Result and Discussion

### 4.1. TGA Analysis

**Figure 1:**
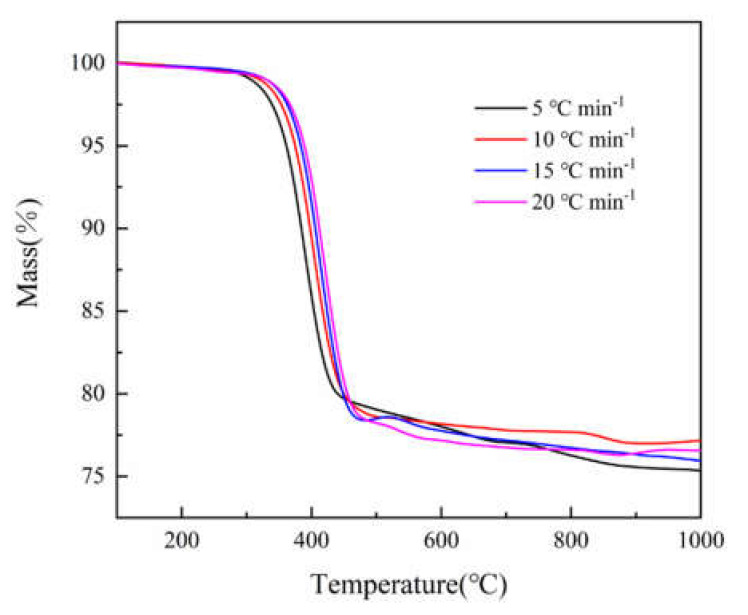
TG curves of glass fiber reinforced plastic at different heating rates (5, 10, 15, 20 °C min^−1^).

**Figure 2:**
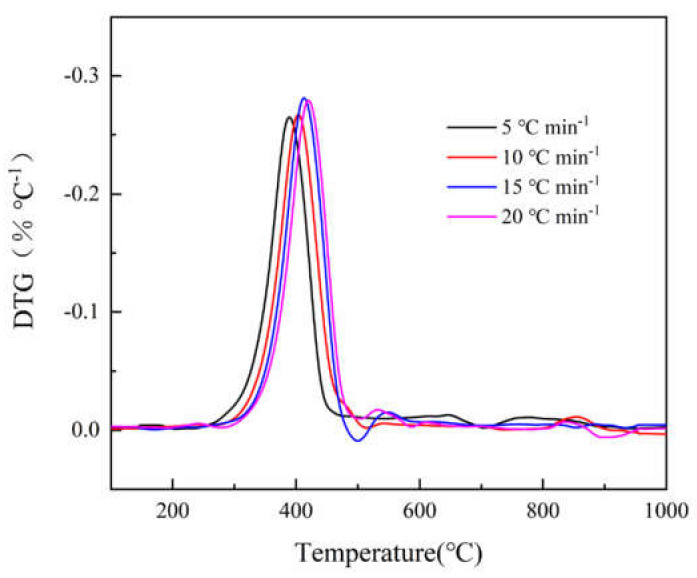
DTG curves of glass fiber reinforced plastic at different heating rates (5, 10, 15, 20 °C min^−1^).

Figure 1 and Figure 2 show the TG and DTG curves of epoxy glass fiber reinforced plastic pyrolysis at different heating rates (5, 10, 15, 20 °C min^−1^). Under different heating rates, the TG and DTG curves have the same variations with increasing temperature, and they both show a shift towards high temperature as the heating rate increases. This is consistent with the law discovered by the researchers mentioned in the introduction [9,10,11,12], indicating that the experimental results of this research are credible. The reason for this phenomenon is when the same temperature is reached, the higher the heating rate, the shorter the reaction time experienced by the sample and the lower the degree of reaction. At the same time, the heating rate affects the heat transfer difference and temperature gradient between the gas phase and the sample surface, which will also affect the heat transfer within solid, resulting in aggravation of thermal hysteresis, causing the curve to shift to the high temperature side. At all different heating rates, the DTG curves have only one main peak during the decomposition of epoxy glass fiber reinforced plastic, and the temperature of the main peak increases as the heating rate increases. The thermal decomposition onset temperature T_0_, the reaction ending temperature T_f_, and the peak temperature T_max_ corresponding to the highest reaction rate are listed in Table 2. It can be observed from Figure 2 that the peak weight loss rate increases significantly as the heating rate increases. The thermal weight loss process of epoxy glass fiber reinforced plastic can be divided into two stages as shown in Figure 1. However, in the second stage, multi-step mass loss occurs when *T* > 460 °C. The cause of this fluctuation may be the unevenness of the sample powder particles. Taking the 5 °C min^−1^ heating rate as an example, the first stage ranges from 290 to 460 °C, during which the TG curve decreases rapidly and the corresponding DTG curve shows a large weight loss peak. The thermal decomposition weight loss rate is 20%, which is the main weight loss stage. The first stage is the thermal decomposition of curing agent acid anhydride and moisture in the epoxy glass fiber reinforced plastic resin material, followed by the thermal decomposition of the epoxy resin matrix. The second stage ranges from 460 to 1000 °C, and then the TG curve decreases gently until the reaction ends, during which stage the weight loss rate is only 5% caused by the further degradation of charring substances generated after the thermal decomposition of the epoxy resin matrix.

### 4.2. Model-Free Analysis

**Figure 3:**
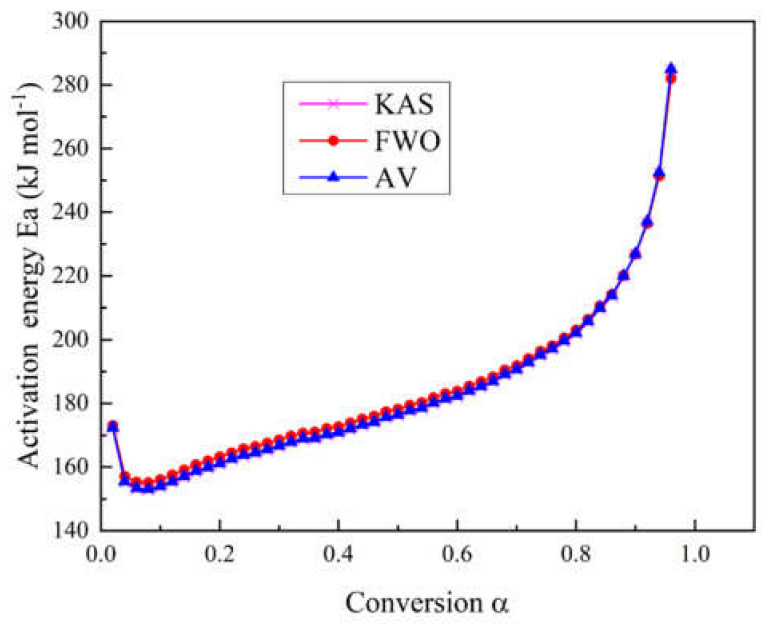
Curves of activation energy calculated with three different model-free methods as a function of conversion rate during the pyrolysis process.

In this study, the KAS method, the FWO method, and the advanced Vyazovkin method were employed to calculate the activation energy of the main pyrolysis process of epoxy glass fiber reinforced plastic. As shown in Figure 3, we can see the dependence of activation energy *Ea* on the conversion rate α during the thermal decomposition of glass fiber reinforced plastics. The variations of the activation energy by the above three methods have maintained a high consistency during the whole process, which can prove the calculation accuracy of the three employed methods.

Regarding the thermal decomposition process of glass fiber reinforced plastic, it can be seen from Figure 3 that although the activation energy values obtained by the three methods are not exactly the same, the trends all decrease first and then increase as the conversion rate increases. Apparently, the whole pyrolysis process can be divided into two stages. In the first stage (290–335 °C) where *α* is 0.02 (*Ea* = 172.4 kJ/mol) to 0.08 (*Ea* = 153.1 kJ/mol), the activation energy decreases with conversion. When *α* = 0.02 and the temperature is close to 300 °C, each molecule shows a polymerized state, and the molecular chain is in a complete state. When the temperature with *α* = 0.08 reaches 335 °C, some of the small molecules, additives, and water in the glass fiber reinforced plastic sample have basically decomposed and are activated. In the second stage with *α* between 0.08–0.96 (335–460 °C), the activation energy increases from 153.1 kJ mol^−1^ to 285 kJ mol^−1^. The main mass loss of this stage is the pyrolysis of the epoxy resin matrix. The results of the three model-free methods in this study show the same tendency with results by Chen and Yeh [14]. The activation energy in [14] shows a monotonously increasing tendency and ranges from 146.44 kJ mol^−1^ to 230.12 kJ mol^−1^.

### 4.3. Model Fitting Analysis

**Figure 4:**
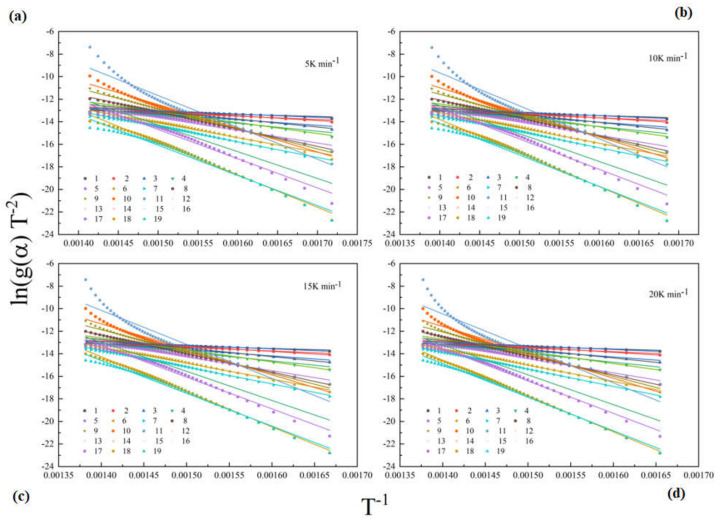
Coats–Redfern (CR) fitting curves of glass fiber reinforced plastic pyrolysis at different heating rates (scatter points represent experimental data for each model, and straight lines represent fitting results) (**a**): 5 K min^−1^; (**b**): 10 K min^−1^; (**c**): 15 K min^−1^; (**d**): 20 K min^−1^.

According to Section 4.2, the model-free method can only obtain part of the kinetic results by calculating the weight loss from TG curves. To obtain the kinetic model *f*(*α*) of epoxy glass fiber reinforced plastic material, model fitting methods are employed in this section. A few researchers have studied the pyrolysis model of the epoxy glass fiber reinforced plastic. During the thermal pyrolysis process of epoxy glass fiber reinforced plastic, we can find that the activation energy in the range of conversion rate 0.02–0.96 changes approximately linearly with the increase in conversion rate. Such a regular variation during the overall degree of conversion means that the reaction is controlled by one single reaction model. Then, the conversion rates are used to fit with different reaction models listed in Table 1. Figure 4 shows the matching results with models listed in Table 1 by the CR method. As shown in Figure 4, the fitting results show a high degree of uncertainty, which indicates that the Arrhenius parameter is strongly dependent on the model selected.

Table 3 lists three models with the best fitting at four different heating rates. For epoxy glass fiber reinforced plastic, first-order (F1), A3/2 (Avrami–Eroféev, n = 1.5), and A2 (Avrami–Eroféev, n = 2) are the three best models selected from all models listed in Table 1. The calculation results of the CR method based on F1 function show good fitting linearity, and the average value of R^2^ can reach 0.9998. Table 3 also lists the linearity of the three models, their activation energy *Ea*, the natural logarithm of the pre-exponential factor ln*A*, and model descriptions with different heating rates. From the model fitting results listed in Table 3, we find that the values of the activation energy *Ea* and pre-exponential factor *A* calculated by the CR method largely depend on the selection of the model, where the value located in an appropriate range identifies a correct selection of the proper pyrolysis model.

### 4.4. Kinetic Compensation Effect

To obtain kinetic triplets through non-isothermal experiments, parameters have a certain dependence on activation energy and pre-exponential factors. Studies have shown that for parameter results of different models, ln*A* has a linear relation with the activation energy *Ea*, which is called the kinetic compensation effect, expressed as,

(13) $$\ln A=a+bE_{a}$$

where *a* = ln*k*_iso_, *b* = 1/*RT*_iso_, *k*_iso_ is the artificial isokinetic rate constant, and *T*_iso_ is the artificial isokinetic temperature. If the model we selected is not proper, the isokinetic temperature value will be located outside of the experimental temperature range. Even if the calculated value of each reaction model included in Table 1 is far from the theoretical value, we can also obtain the corresponding activation energy and pre-exponential factor value of each reaction model by calculation. Different reaction models can be used to calculate and obtain corresponding triplets, and the pre-exponential factor can be predicted as a function of the activation energy value through the kinetic compensation effect.

**Figure 5:**
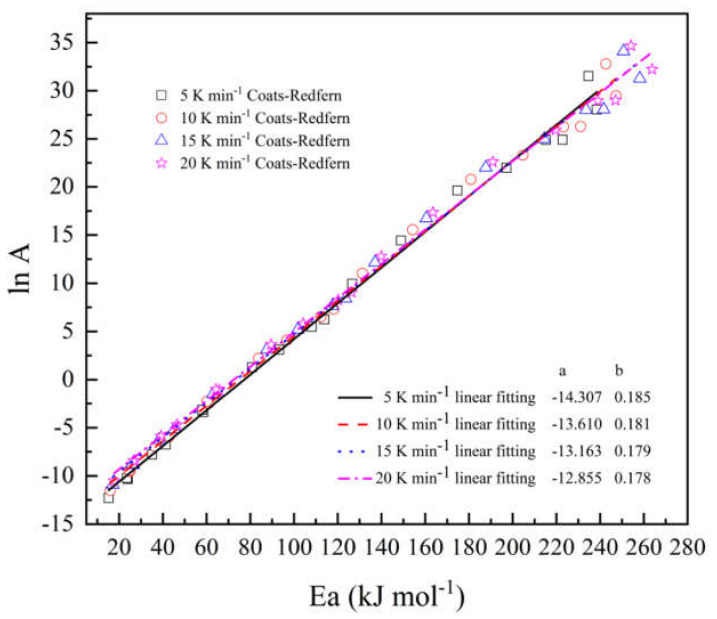
Linear relationship between the natural logarithm of the pre-exponential factor (ln*A*) and activation energy (*Ea*) at different heating rates.

**Figure 6:**
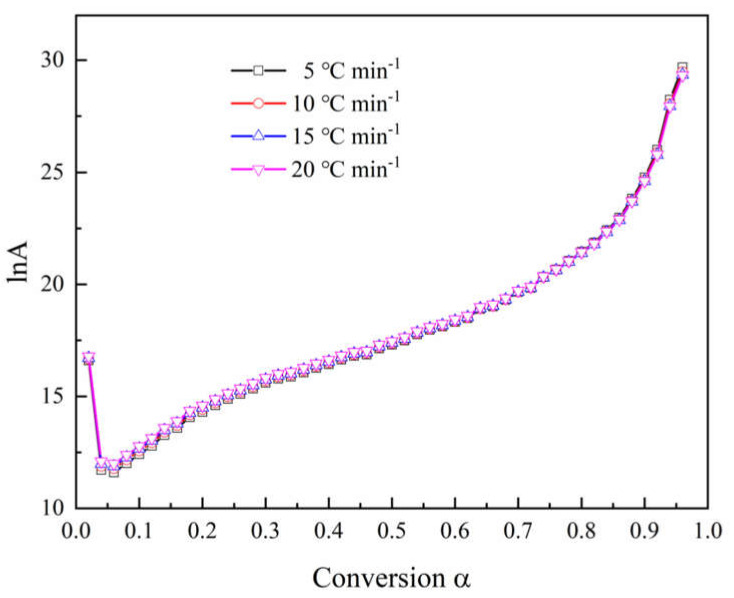
Relationship between the natural logarithm ln*A* of the pre-exponential factor and the conversion rate *α* at different heating rates.

Figure 5 indicates the compensation effect diagram for thermal decomposition of the epoxy glass fiber reinforced plastic, which was obtained by using the CR method at four different heating rates. As the heating rate increases, the temperature of artificial isokinetics will increase as well. When the reaction model is selected properly, the artificial isokinetic temperature will be exactly located at the experimental temperature range. From Figure 5, we can see that the kinetic triplets of all models show good linearity, and the artificial isokinetic temperature values are located in the temperature range of the experiment, and this proves that the proper model has been selected. As shown in Figure 6, then the pre-exponential factor at each conversional extent can be obtained.

### 4.5. FTIR Analysis

**Figure 7:**
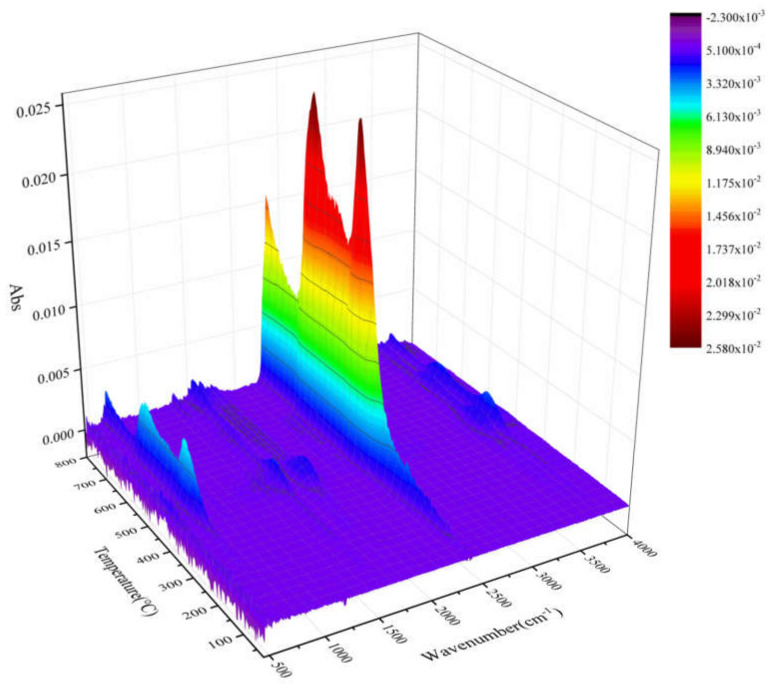
The 3D emission gas spectra of glass fiber reinforced plastic at different temperatures.

**Figure 8:**
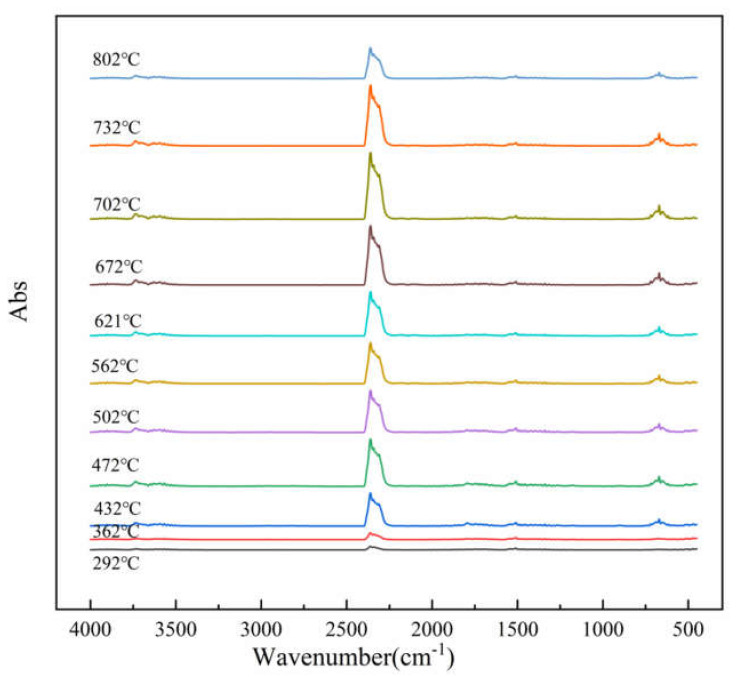
FTIR spectrum of thermal degradation of glass fiber reinforced plastic at a heating rate of 20 °C min^−1^.

**Figure 9:**
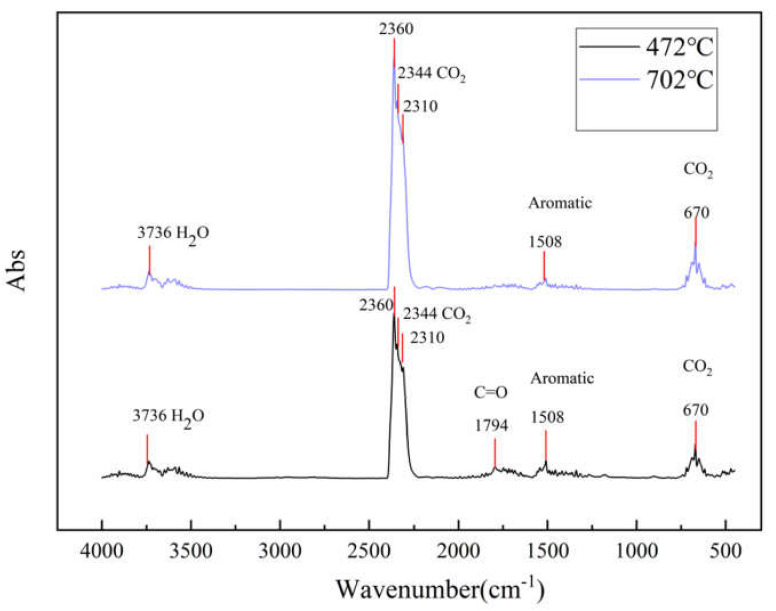
FTIR spectra of glass fiber reinforced plastic at 472 °C and 702 °C.

TG–FTIR uses a specific purge gas (usually nitrogen, air or helium) to pass the volatile or decomposition products generated during the weight loss process through metal pipes and glass at a constant high temperature (usually 200–250 °C). A gas cell is introduced into the optical path of an infrared spectrometer to perform infrared detection and analyze and judge the structure of the outgas components. FTIR spectra of evolved gas during pyrolysis ranging from 100 to 800 °C in a nitrogen atmosphere are shown in Figure 7. Figure 8 shows a clearer infrared spectrum of epoxy glass fiber reinforced plastic at different temperatures. As shown in Figure 8, the main wavenumber ranges of the absorption peak are 4000–3500, 2400–2200, 1900–1300, and 800–600 cm^−1^. Where the intensity of the absorption peak is 702 °C, it reaches the maximum and appears in the wavenumber range of 2400~2200 cm^−1^. Figure 9 shows FTIR spectra of escaped volatiles of glass fiber reinforced plastic at 472 and 702 °C. We can see that the main volatile components of epoxy glass fiber reinforced plastic are identified as H_2_O (3736 cm^−1^) [34], CO_2_ (2360, 2344, 2310, and 670 cm^−1^), carbonyl components (1794 cm^−1^), and aromatic components (1510 cm^−1^).

**Figure 10:**
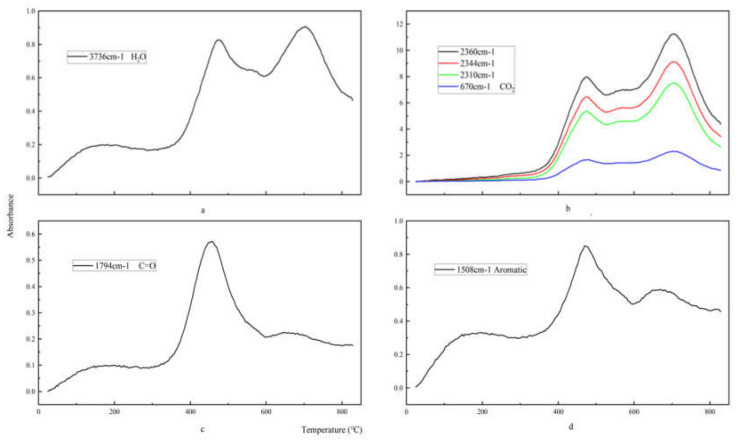
The density distribution of H_2_O, CO_2_, carbonyl, and aromatic products of glass fiber reinforced plastic. The density distribution of H_2_O (**a**), CO_2_ (**b**), carbonyl (**c**), and aromatic products (**d**) of glass fiber reinforced plastic.

Figure 10 shows the intensity curve of each characteristic absorption peak as a function of temperature. As shown in Figure 10a,b, the characteristic absorption peaks of the gas products H_2_O and CO_2_ of epoxy glass fiber reinforced plastic have two peaks, which appear at about 472 and 702 °C, respectively. Both peaks reach the maximum value at 702 °C, with the same trend as the total amount of gas released by the epoxy glass fiber reinforced plastic samples pyrolysis process. However, the volatile product carbonyl and aromatic compound benzene in Figure 10c show a significant peak at 472 °C, and the subsequent peaks are not obvious. The reasons for this can be explained by the first stage of pyrolysis, where the epoxy resin matrix main chain of the epoxy glass fiber reinforced plastic sample is broken and the small molecule curing agent acid anhydride is volatilized to generate carbonyl groups, H_2_O, CO_2_ and aromatic benzene. When the temperature continues to rise, the second phase of pyrolysis begins, and the characteristic peak intensities of carbonyl and volatile macromolecular benzene decrease (as shown in Figure 10c,d), indicating that the carbonyl and benzene ring have reacted, and thus forming large amount of H_2_O and CO_2_. In addition, water is produced in three ways. First, the water in the sample begins to evaporate when the temperature reaches 100 °C. This process corresponds to the shoulder peak shown in Figure 10a. Secondly, the primary alcohol on the epoxy resin dehydrates to form water, and this can be found from the first peak shown in Figure 10a. Then, the second peak of water explains the decomposition of the benzene.

**Figure 11:**
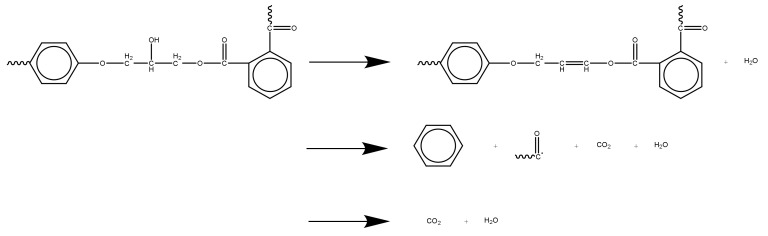
The chemical reaction mechanism of glass fiber reinforced plastic.

As the epoxy glass fiber reinforced plastic sample is pyrolyzed mainly in the epoxy resin matrix within the testing temperature range, the glass fiber itself does not decompose. In summary, the reaction mechanism of epoxy glass fiber reinforced plastic, that is, the reaction mechanism of epoxy resin after curing, is shown in Figure 11. The first is the dehydration of the primary alcohol on the epoxy resin. Secondly, the epoxy resin matrix main chain of the glass fiber reinforced plastic sample breaks, and the small molecule curing agent acid anhydride volatilizes to generate carbonyl groups and a large amount of H_2_O, CO_2_, and aromatic benzene. When the temperature continues to rise, it enters the second stage of pyrolysis, where the carbonyl group reacts with volatile macromolecular benzene to form a large amount of H_2_O and CO_2_.

## 5. Conclusions

Epoxy glass fiber reinforced plastics have been used more and more widely in various fields due to their superior performance. However, there are few studies on the thermal decomposition kinetics of glass fiber composites, especially their kinetics models and reaction mechanism. This paper conducts pyrolysis kinetics and reaction mechanism research on epoxy glass fiber reinforced plastic with TG and TG–FTIR technologies. The obtained pyrolysis kinetic parameters can provide useful reference data on thermal degradation, thermal aging, and other thermal properties of epoxy glass fiber reinforced plastic. This paper gives a complete analysis method of the kinetics and the pyrolysis mechanism of epoxy glass fiber reinforced plastics, and the method can also be applied to other polymer materials to obtain their kinetic information and pyrolysis reaction mechanism. This paper provides kinetic parameters for modellings of thermal ablation, combustion, and thermal aging of epoxy glass fiber reinforced plastics materials. Relevant conclusions are as follows:

(1) The pyrolysis process of epoxy glass fiber reinforced plastic in a nitrogen atmosphere can be roughly divided into two stages. During the pyrolysis stage, the main volatile products are H_2_O (3736 cm^−1^), CO_2_ (2360, 2344, 2310 and 670 cm^−1^), carbonyl components (1794 cm^−1^), and aromatic components (1510 cm^−1^). The first stage corresponds to the temperature range of 290–460 °C, and the weight loss is about 20%. In the first stage, the main chain of the additives and epoxy resin matrix in the glass fiber reinforced plastic sample were broken and decomposed into small molecules such as H_2_O and CO_2_ gas and a large amount of volatile macromolecular benzene. The second stage corresponds to 460–1000 °C and the weight loss is about 5%. In the second stage, the volatile macromolecular benzene is further decomposed to generate a large amount of H_2_O and CO_2_.
(2) Model-free and model fitting methods were employed to calculate kinetic parameters of epoxy glass fiber reinforced plastic. Through the model-free method, we found that the activation energy *Ea* of epoxy glass fiber reinforced plastic decreased firstly and then increased with the conversion rate *α*. When 0.02 < *α* < 0.08, *Ea* decreases from 172.4 to 153.1 kJ mol^−1^. When 0.08 < *α* < 0.96, *Ea* increases from 153.1 to 285 kJ mol^−1^. Through the model fitting method, we found that the three models with the best fitting under four different heating rates are first-order (F1), A3/2 (Avrami–Eroféev (n = 1.5)), and A2 (Avrami–Eroféev (n = 2)), respectively.

## Figures and Tables

**Table 1:** A set of reaction models describing the pyrolysis process of matter. *f*(*α*) is the pyrolysis model in Arrhenius equation, and *g*(*α*) is the integral form of *f*(*α*).

Reaction Model	Model Code	*f*(*α*)	*g*(*α*)
P1	Power law	4*α*^3/4^	*α* ^1/4^
P2	Power law	3*α*^2/3^	*α* ^1/3^
P3	Power law	2*α*^1/2^	*α* ^1/2^
P4	Power law	2/3*α*^−1/2^	*α* ^3/2^
R1	Zero-order (Polany-Winger Equation)	1	*α*
R2	Phase-boundary controlled reaction	2(1 − *α*)^1/2^	[1 − (1 − *α*)^1/2^]
R3	Phase-boundary controlled reaction	3(1 − *α*)^2/3^	[1 − (1 − *α*)^1/3^]
F1	First-order	(1 − *α*)	−ln(1 − *α*)
F3/2	Three-halves order	(1 − *α*)^3/2^	2[(1 − *α*)^−1/2^ − 1]
F2	Second-order	(1 − *α*)^2^	(1 − *α*)^−1^ − 1
F3	Third-order	(1 − *α*)^3^	(1/2)[(1 − *α*)^−2^ − 1]
A3/2	Avrami–Eroféev (n = 1.5)	(3/2)(1−*α*)[−ln(1 − *α*)]^1/3^	[−ln(1 − *α*)]^2/3^
A2	Avrami–Eroféev (n = 2)	2(1 − *α*)[−ln(1 − *α*)]^1/2^	[−ln(1 − *α*)]^1/2^
A3	Avrami–Eroféev (n = 3)	3(1 − *α*)[−ln(1 − *α*)]^2/3^	[−ln(1 − *α*)]^1/3^
A4	Avrami–Eroféev (n = 4)	4(1 − *α*)[−ln(1 − *α*)]^3/4^	[−ln(1 − *α*)]^1/4^
D1	One-dimensional diffusion	1/2*α*	*α* ^2^
D2	Two-dimensional diffusion	1/[−ln(1 − *α*)]	(1 − *α*)ln(1 − *α*) + *α*
D3	Three-dimensional diffusion	3(1 − *α*)^1/3^/2[(1 − *α*)−1/3 − 1]	[1 − (1 − *αα*)^1/3^]^2^
D4	Three-dimensional diffusion	3/2[(1 − *α*)^−1/3^ − 1]	(1 − 2*α*/3) − (1 − *α*)^2/3^

**Table 2:** Characteristic temperature values of the decomposition process obtained by TGA curve with different heating rates.

β (°C min^−1^)	T_0_ (°C)	T_max_ (°C)	T_f_ (°C)
5	234.9	388.6	479.6
10	253.5	403.9	520.9
15	257.2	412.2	502.1
20	273.9	421.5	513.7

**Table 3:** The three best models and their parameters with fitting parameters for the pyrolysis process obtained by the Coats–Redfern method.

	5 °C min^−1^				10 °C min^−1^				15 °C min^−1^				20 °C min^−1^			
	Model	ln*A*	*Ea*	R^2^	Model	ln*A*	*Ea*	R^2^	Model	ln*A*	*Ea*	R^2^	Model	ln*A*	*Ea*	R^2^
glass fiber reinforced plastic	F1	9.94	126.61	0.9985	F1	11.01	131.31	0.9991	F1	12.16	137.04	0.9998	F1	12.78	139.90	0.9993
	A3/2	1.28	80.83	0.9983	A3/2	2.22	83.89	0.9990	A3/2	3.13	87.67	0.9998	A3/2	3.65	89.56	0.9992
	A2	−3.16	57.94	0.9980	A2	−2.28	60.18	0.9988	A2	−1.49	62.98	0.9998	A2	−1.03	64.38	0.9992
